# Dramatic clinical response to dabrafenib plus trametinib in anaplastic thyroid carcinoma and the challenges faced during the COVID-19 pandemic

**DOI:** 10.20945/2359-3997000000325

**Published:** 2021-02-15

**Authors:** Fernanda Bueno, Erika Abelleira, Florencia von Stecher, Andrea Paes de Lima, Fabián Pitoia

**Affiliations:** 1 University of Buenos Aires Division of Endocrinology Buenos Aires Argentina Division of Endocrinology, University of Buenos Aires, Buenos Aires, Argentina; 2 University of Buenos Aires Department of Pathology Buenos Aires Argentina Department of Pathology, University of Buenos Aires, Buenos Aires, Argentina

## Abstract

Anaplastic thyroid carcinoma is the rarest tumor of the thyroid gland, representing less than 2% of clinically recognized thyroid cancers. Typically, it has an extremely rapid onset, fatal outcomes in most cases, and a median overall survival of 3 to 10 months despite aggressive multidisciplinary management. The presence of targetable mutations in anaplastic thyroid carcinoma patients is an opportunity for treatment when conventional therapeutics approaches are not effective, a frequent situation in the majority of patients. We present our experience in the management of a patient with unresectable anaplastic thyroid cancer who had a remarkable and rapid response to treatment with dabrafenib and trametinib during the COVID-19 pandemic. After four weeks of dabrafenib 150 mg twice daily plus trametinib 2 mg daily, he showed a dramatic reduction of the cervical mass around 90%. Nearly eight weeks under treatment with dabrafenib plus trametinib, the patient remains with minimal locoregional disease without distant metastases.

## INTRODUCTION

Anaplastic thyroid carcinoma (ATC) is the rarest tumor of the thyroid gland, representing less than 2% of clinically recognized thyroid cancers (1,2). Typically, it has an extremely rapid onset and fatal outcomes in most cases, accounting for more than half of all deaths attributable to thyroid tumors (3). At the time of diagnosis, most cases are associated with extensive local disease, and the presence of distant metastasis is observed in 20% to 50% of patients (4). The most common metastatic sites are the lungs, followed by intrathoracic and neck lymph nodes (5). Until 2018, ATC was associated with a median overall survival (OS) of 3 to 10 months, a median 1-year survival of 20%, and a median 10-year OS of less than 2%, despite aggressive multidisciplinary management (6–11). The most recent database analysis reported ATC was associated with an improvement in median OS in the last three years (12).

ATC's locoregional invasiveness may cause compressive symptoms, such as dysphagia, dyspnea, stridor, and pain, which generally make ATC unresectable. Therefore, the diagnostic and therapeutic management of this entity represents a challenging situation, and a multidisciplinary team with experience in ATC management is always required (13).

According to the American Joint Committee on Cancer, ATC is classified as Stage IV, regardless of tumor size or the presence of lymph node or distant metastasis: Stage IVA describes all tumors confined to the gland; Stage IVB represents ATC with gross extrathyroidal extension; and in Stage IVC, the tumor has already spread to distant sites (14,15).

The American Thyroid Association's (ATA) guidelines consider surgical resection, when feasible, as first-line treatment and external beam radiation therapy for local control (16). ATC is a rare and aggressive tumor, thus predicting patient clinical therapy responsiveness is still challenging. Several genetic mutations have been described in ATC, that are involved in different molecular pathways linked to tumor progression, and novel therapies acting on these molecular pathways have been investigated to improve the quality of life in these patients (15,17,18). Mutation of the tumor suppressor p53 gene is commonly detected among ATC (70–88%), whereas the gene is found less frequently in follicular thyroid cancer and papillary thyroid cancer (PTC) (19). Alternatively, approximately 20–50% of anaplastic thyroid tumors harbor the *BRAF (V600E)* mutation (20–23).

To assess the efficacy and safety of *BRAF* plus *MEK* inhibition, an open-label Phase II trial evaluated dabrafenib plus trametinib in patients with nine rare tumor types, including patients with ATC. The objective response rate (ORR) was 67%, including two complete responses. In the subset of patients with centrally confirmed *BRAF (V600E)* mutation-positive tumors, the ORR was 75%. The median progression-free survival (PFS) was 13.8 months, and the median OS was 19.8 months (24). This multicenter phase II trial led to approval by the United States Food and Drug Administration in May 2018 for this combination of drugs for patients with *BRAF (V600E)*-mutated ATC with locally advanced, unresectable, or metastatic ATC with no locoregional treatment options.

The presence of targetable mutations in ATC patients is an opportunity for treatment when conventional therapeutics approaches are not effective, a frequent situation in the majority of patients (25). However, the management of these patients represents a challenge in Latin America, considering the underlying resource limitations of the health system, whether for the access to genomic testing or the subsequent targeted therapy. We previously reported how the advances in this scenario changed the outcomes. In our cohort of advanced thyroid cancer with targetable mutations, only 32% of patients who were offered the genomic test could afford to pay for the test or have the test covered by their health insurance (26,27). Considering the three patients with unresectable *BRAF (V600E)* positive ATC, two patients received dabrafenib plus trametinib (D-T) with significant clinical benefits, but one patient died due to rapidly progressive disease before health insurance authorized D-T (27).

In this paper, we present our experience in the management of a patient with unresectable ATC with a remarkable and rapid response to treatment with D-T during the COVID-19 pandemic.

## CLINICAL CASE

A 62-year-old man with a 5-year history of a locally advanced persistent PTC was referred to our hospital in March 2020 with a rapidly growing cervical mass. He underwent total thyroidectomy and remnant ablation in 2015. The same year, he received an additional therapeutic radioiodine dose of 250 mCi. In June 2018, he evolved with a structural incomplete response in the left lateral neck region, with fine-needle aspiration confirming a metastatic papillary thyroid carcinoma. The patient discontinued the follow-up for one year. Four years after the initial diagnosis (July 2019), he underwent a new surgery for a 45 × 35 × 20 mm cervical lesion (VI level), with incomplete resection due to tracheal adhesion. In August 2019, he received the third therapeutic dose of radioactive iodine (150 mCi), with a post-dose whole-body scan showing only a faint uptake in the right lateral neck. Subsequently, the patient developed a rapidly growing cervical mass 2 months before he was referred to our hospital.

**Figure 1:**
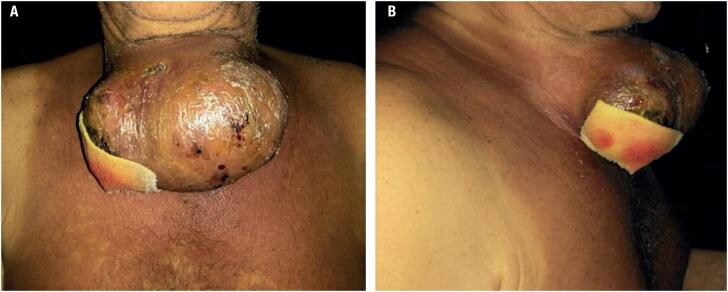
(**A** and **B**): Pictures of the patient's neck in the first clinical examination (large anterior cervical mass). Generalized inflammation (lymphangitis) was noted around the mass and extending to the anterior thoracic region.

On clinical examination, a mass was palpated at the anterior cervical region, with increased consistency, a smooth surface, and adherence to the deep planes, including generalized inflammation (lymphangitis) around the mass and extending to the anterior thoracic region (Figures 1A and B). Vital signs were normal. He mentioned dysphagia to solid food. He denied fever, dyspnea, or other symptoms of local compression.

## COMPLEMENTARY EXAMINATIONS

**Figure 2:**
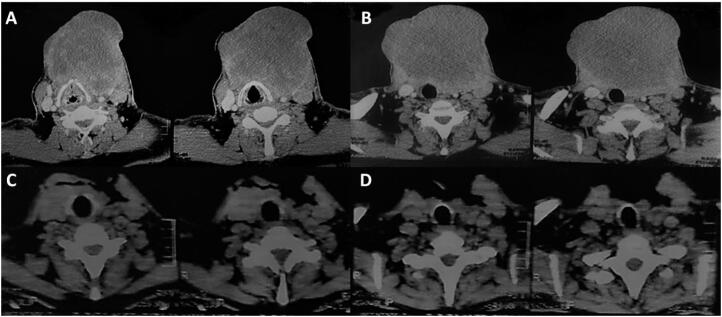
(**A** and **B**): Axial view of Computed Tomography (CT) scans of the neck showing the cervical mass before treatment: 120 × 130 mm in the thyroid and left lateral neck. (**C** and **D**): Four weeks after treatment, the axial view of CT scans showed a dramatic reduction of the cervical mass around 90%.

His baseline investigations were normal, including thyroid function tests. Infection and microbiology workup all came out as negative. Computed tomography (CT) scans of the neck, chest, and abdomen were performed. The neck CT revealed an unresectable lesion of 130 × 120 mm (APxT) involving the trachea, esophagus, carotid artery, jugular vein, and extending to the thoracic inlet (Figures 2A and B). This lesion measured 62 × 42 mm on a CT scan performed 2 months before the patient's admission at our institution.

**Figure 3:**
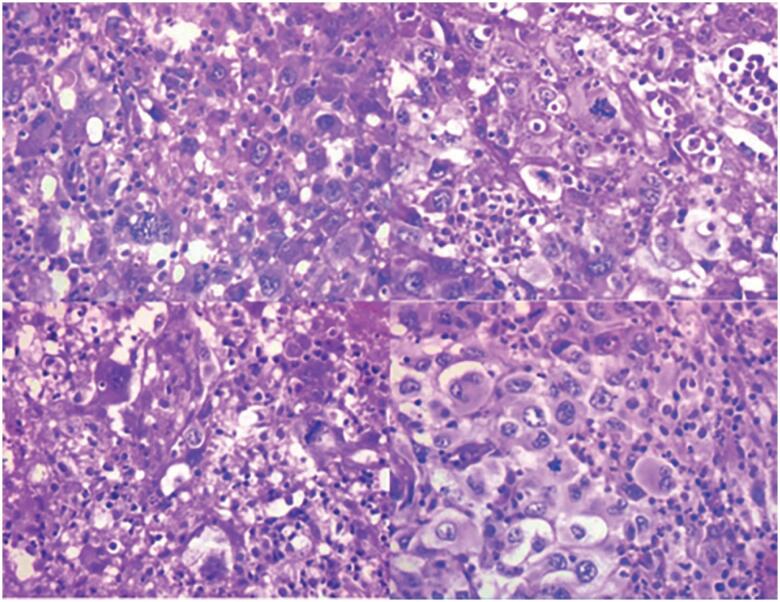
ATC. Pleomorphic tumor cells with atypical mitosis admixed with neutrophils (hematoxylin-eosin, X400).

The cervical lesion was biopsied by fine-needle aspiration and revealed neoplastic cells, with marked nuclear pleomorphism, macrokaryosis, evident nucleoli multinucleation, isolated bizarre nuclei, and moderate cytoplasm in a background of lymphocytes and neutrophils. However, the material was not suitable for the molecular study. Therefore, an excisional biopsy was performed, which showed an undifferentiated malignancy with pleomorphic cells in a diffuse pattern, bizarre nuclei, and multinucleation. Mitosis was frequent, and extensive areas of necrosis, hemorrhage and suppuration were observed (Figure 3). The biopsy finally confirmed the presence of ATC. ATC

He was tested for the *BRAF* status through next-generation sequencing on a tumor tissue sample, confirming the presence of a *BRAF (V600E)* mutation.

### Outcome and follow-up

The tumor was considered unresectable due to tracheal involvement and extensive disease surrounding the carotid; he was not a candidate for primary surgical resection.

As D-T was not immediately available, *off-label* lenvatinib 10 mg/day was used as *bridging* therapy. Ten days later, the development of bleeding, minimal ulceration in the lesion, and fever made it necessary to withdraw lenvatinib treatment. In the context of the COVID-19 pandemic, the patient was swabbed to rule out a SARS-CoV-2 infection.

**Figure 4:**
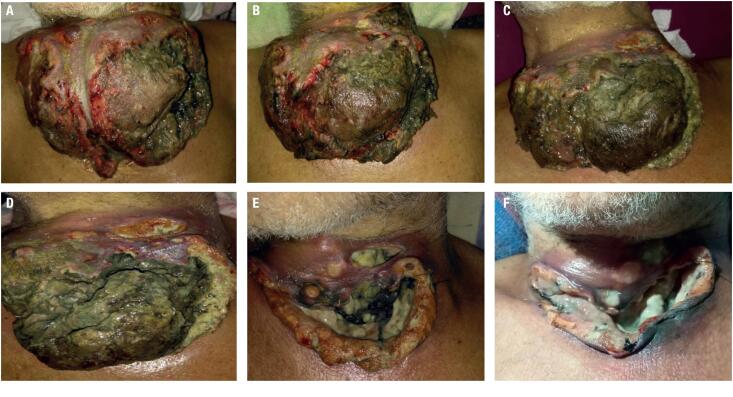
Lesion outcome under treatment with D-T. **A:** before treatment. **B:** one day under treatment. **C:** four days under treatment. **D:** seven days under treatment. **E:** ten days under treatment. **F:** four weeks under treatment.

On June 2, the patient began the combination treatment: dabrafenib 150 mg twice daily plus trametinib 2 mg daily. At 48 hours, the patient evolved with improvement in cervical pain and discontinued opioid intake. At 72 hours, there was evidence of tumor lesion necrosis. After only one week of therapy, the patient had a marked reduction of approximately 50%, with a total resolution of dysphagia. The cervical lesion evolved with extensive necrosis and tracheal exposure; the patient was reevaluated for oncological surgery and otorhinolaryngology service, which indicated flat cures (Figure 4).

During the treatment with D-T, the patient presented adverse effects: (i) hyperglycemia (blood glucose level of 235 mg/dl, before treatment blood glucose levels ranged from 110 to 124 mg/dL), for which reason he was evaluated by the Diabetes Care Unit, they indicated dietary treatment and started treatment with metformin; (ii) fatigue grade 2; and (iii) constipation. The patient had a performance status grade 2.

Additionally, the patient presented fever associated with odynophagia, which led us to rule out SARS-CoV-2 several times during the treatment with D-T. Finally, the fever resolved with physical methods and antipyretic drugs, and we considered the fever as an adverse effect of D-T treatment.

Four weeks after treatment, an axial view of neck CT scans showed a dramatic reduction of the cervical mass of approximately 90% (Figures 2C and D). The patient remains with a minimal locoregional disease without distant metastases, nearly eight weeks under treatment with D-T.

## DISCUSSION

The aggressive behavior of ATC confers a rapidly fatal outcome. The immediate cause of death in most patients is related to local complications, such as airway obstruction, catastrophic hemorrhage, or circulatory failure due to compression of mediastinal vasculature (9,10). Locally advanced disease also leads to severe pain and dysphagia. In a limited subset of patients with resectable disease at presentation, surgical resection has been associated with improved survival. However, for most patients who present with advanced T4b disease, radical resection is associated with increased morbidity and is not advocated because of rapid recurrence, distant disease, and poor survival outcomes.

*BRAF (V600E)* point-activating mutation occurs in approximately 20–50% of ATC (20–23). Additionally, other targetable mutations have been found in ATC: *RET/PTC* rearrangements and *N-TRK* rearrangements, which led to therapeutical success in isolated case reports (28,29).

Fortunately, the therapeutic management of ATC has expanded with the appearance of the first combination of selective *BRAF* plus *MEK* inhibitor therapy approved for mutated ATC *BRAF (V600E)*, with high response rates and a significant improvement in survival (24). Some patients with initially unresectable disease may become resectable and could be considered for surgery if they respond to anti *BRAF*/*MEK* combination therapy. Wang and cols. reported the first series in the literature of *BRAF (V600E)*-mutated ATC patients with locoregionally advanced disease treated with DT followed by surgical resection. They demonstrated the feasibility for complete tumoral resection, decreased need for tracheostomy, high pathologic response rates, and durable locoregional control (30). We reported a patient with locally advanced and metastatic anaplastic thyroid carcinoma with a *BRAF (V600E)* mutation with complete surgical resection after D-T treatment (26).

A recently published retrospective cohort study included 479 patients with ATC from January 2000 to October 2019, which were divided into three groups according to the date of presentation: 2000–2013, 2014–2016, and 2017–2019. The median OS of the entire cohort was 9.5 months. The OS at 2 years was 18% in the 2000–2013 group, and 42% in the 2017–2019 group, respectively (*P* < .001). Patients undergoing surgery following neoadjuvant *BRAF*-directed therapy had a 94% 1-year survival, with a median follow-up of 1.21 years. They concluded that the changes in patient management, such as molecular-based personalized therapies, appear to be associated with a significant increase in survival (12).

A multidisciplinary team with experience in ATC must carry out the diagnostic and therapeutic management of this entity. This situation represents a challenge in Latin American considering the underlying resource limitations. On the other hand, the management of this patient was more difficult in the context of the COVID-19 pandemic. Thus, in the presence of fever on two occasions, the viral infection had to be ruled out.

This is a report that contributes to illustrate the feasibility and effectiveness of a neoadjuvant approach using D-T in one patient with locoregionally advanced *BRAF (V600E)*-mutated ATC. Further studies are required to evaluate systematically the effect of this strategy on overall survival, progression-free survival, and quality of life.
